# Early vitamin AD supplementation reduces transfusion needs in preterm infants: a retrospective cohort study

**DOI:** 10.3389/fnut.2026.1711120

**Published:** 2026-03-27

**Authors:** Futao Li, Xuhui Ye, Pan Tian, Ping Zhou, Xintian Shen

**Affiliations:** 1 Department of Pharmacy, Shenzhen Baoan Women's and Children's Hospital, Shenzhen, Guangdong, China; 2 Department of Neonatology, Shenzhen Baoan Women's and Children's Hospital, Shenzhen, Guangdong, China

**Keywords:** anemia of prematurity, blood transfusion, preterm infants, risk factors, vitamin A, vitamin D

## Abstract

**Introduction:**

Anemia of prematurity (AOP) is a common condition, often necessitating transfusion therapy. Although vitamin AD deficiency has been linked to anemia pathogenesis, the effect of supplementation timing on transfusion needs remains unclear. This study investigates the association between the initiation timing of vitamin AD supplementation and the number of transfusions received in preterm infants.

**Methods:**

In this retrospective cohort study, we included 226 preterm infants (gestational age <37 weeks) who received both blood transfusions and vitamin AD supplementation in a neonatal intensive care unit (2018–2022). The association was assessed using Spearman correlation. Segmented Poisson regression compared transfusion incidence rates before and after supplementation, controlling for hospitalization timeline. A conditional Cox model (Anderson-Gill) evaluated the impact on subsequent transfusion risk, adjusting for covariates. Patients were stratified by supplementation timing (≤14 vs. >14 days), with propensity score matching applied to balance groups. Independent factors were identified using generalized linear models.

**Results:**

The timing of vitamin AD initiation was positively correlated with transfusion number [Spearman’s *ρ* = 0.200(0.068–0.326), *p* = 0.003], an association that remained significant in subgroups including infants 28–37 weeks and those without complications. Multivariable analysis identified supplementation timing as an independent risk factor for transfusion frequency [*β* = 0.016(0.007–0.025), *p* < 0.001]. Segmented Poisson regression showed a significantly lower incidence rate ratio after supplementation [IRR = 0.139(0.098–0.199)]. The conditional Cox model revealed no immediate change in risk [*HR* = 10.858(0.725–162.663), *p* = 0.084] but a significant time-dependent protective effect [*HR* per log(day+1) = 0.381(0.160–0.911), *p* = 0.03], with the hazard ratio becoming protective by day 5 and reaching 0.32 (68% risk reduction) by day 28. After propensity score matching, no significant difference in transfusion frequency was found between the ≤14-day and >14-day groups [median (IQR): 2 (1.5–3) vs. 2 (2–4), *p* = 0.544].

**Conclusion:**

This study suggests an association between earlier vitamin AD supplementation and reduced transfusion needs in preterm infants, supported by a time-dependent protective effect. The specific optimal timing requires further prospective investigation.

## 1 Introduction

Anemia is a common clinical complication in neonates, particularly among preterm infants. Due to immaturity of various organ systems, the hematopoietic system in preterm infants is characterized by reduced erythropoietin (EPO) production, shortened red blood cell lifespan, and/or impaired bone marrow erythropoiesis, predisposing them to anemia of prematurity (AOP) (1). AOP significantly hampers normal growth and development and may increase the risk of long-term adverse outcomes such as neurodevelopmental impairment (2). The incidence of AOP is markedly higher in preterm infants (gestational age <37 weeks). Epidemiological data indicate that up to 80% of very low birth weight infants (VLBWI) and 95% of extremely low birth weight infants (ELBWI) require transfusion during hospitalization (3).

Vitamin A, which is primarily stored in the liver, blood, and retina, plays vital biological roles including promoting growth, maintaining epithelial integrity, participating in rhodopsin synthesis, and exerting antioxidant effects (4). Preterm infants are particularly susceptible to vitamin A deficiency (5, 6), which increases their risk of infections, growth retardation, and xerophthalmia (7). Vitamin D is mainly involved in regulating calcium and phosphorus metabolism and maintaining serum levels of both minerals. It also exhibits anti-proliferative and differentiation-inducing effects on tumor cells, promotes TNF release by macrophages, inhibits the growth of colon cancer and melanoma, and contributes to immune regulation and homeostasis (8). Studies have shown that most preterm infants are vitamin D deficient at birth (9), which is associated with respiratory distress syndrome, necrotizing enterocolitis, bronchopulmonary dysplasia, and metabolic bone disease (10). In the context of anemia, vitamin A deficiency may inhibit EPO expression and disrupt erythropoiesis (11), while vitamin D can directly suppress hepcidin mRNA transcription or indirectly downregulate hepcidin by inhibiting inflammatory cytokines. It also acts synergistically with EPO to promote the proliferation of erythroid progenitor cells, thereby participating in hematopoietic regulation (12, 13).

Although combined vitamin A and D supplementation (as vitamin AD) is routinely administered to neonates, there is considerable variation in the timing of initiation. The relationship between the timing of initial vitamin AD supplementation and transfusion outcomes—such as transfusion frequency—remains inadequately studied. Therefore, this retrospective study aims to investigate the association between the timing of vitamin AD initiation and the number of transfusions received in preterm infants.

## 2 Materials and methods

### 2.1 Study population

This retrospective cohort study included preterm infants admitted to the neonatal intensive care unit (NICU) of our hospital between October 2018 and December 2022. The inclusion criteria were as follows: gestational age < 37 weeks; hospitalization in the NICU for ≥ 7 days after birth; receipt of blood transfusion and oral vitamin AD supplementation during hospitalization; and availability of complete clinical records, including documentation of vitamin AD supplementation, transfusion history, and related complications. Infants were excluded if they had major congenital anomalies, inherited metabolic diseases, maternal severe complications during pregnancy that affected fetal development, or incomplete medical records.

### 2.2 Data collection

The clinical data collected for preterm infants included: baseline characteristics [sex, birth weight (BW), gestational age (GA), mechanical ventilation (MV), plurality, mode of delivery], occurrence of complications [patent ductus arteriosus (PDA), small for gestational age (SGA), feeding intolerance (FI), intracranial hemorrhage (ICH), necrotizing enterocolitis (NEC)], laboratory and treatment parameters [hemoglobin (Hb) concentration and hematocrit (HCT) at birth, time to initiation of breastfeeding, time to initiation and dosage of oral iron supplementation, time to achieve full enteral feeding, number of doses and total dosage of erythropoietin administered during hospitalization], as well as the average and peak serum 25-hydroxyvitamin D levels monitored during the hospital stay.

The primary study measures included: (1) Timing of supplementation: defined as the time to initiation of vitamin AD drops and vitamin D drops (both calculated as the number of days from birth to first supplementation), with the vitamin D lead time calculated as the number of days by which vitamin D supplementation preceded vitamin AD supplementation; (2) Transfusion outcomes: including the total number of transfusions during hospitalization and the specific timing of each transfusion event. Furthermore, the study documented the occurrence of major complications during hospitalization [such as sepsis, retinopathy of prematurity (ROP), bronchopulmonary dysplasia (BPD), gastrointestinal (GI) hemorrhage, and cholestasis] to assess their potential influence as confounding factors.

All patient data were obtained from medical records. The study protocol was approved by the Ethics Committee of Shenzhen Baoan Women’s and Children’s Hospital. All data were de-identified prior to analysis.

### 2.3 Standardized anemia management protocol

Our department has established a standardized anemia management protocol for preterm infants, which includes the following key components:

1. Delayed cord clamping for at least 30 s in preterm neonates who do not require immediate resuscitation.
2. Collection of sufficient umbilical cord blood from very preterm and extremely preterm infants for routine laboratory testing after birth.
3. Minimization of blood sampling by adopting micro-sample techniques for routine tests and extending the intervals between tests when the infant’s condition is stable.
4. Initiation of subcutaneous erythropoietin (EPO) at 1 week of age, followed by iron supplementation (elemental iron 2–4 mg/kg/day) starting from 2 weeks of age.

### 2.4 Transfusion protocol

Referring to both domestic and international guidelines, such as China’s health industry standard Guideline for Pediatric Transfusion, our department has developed its own transfusion protocol. This protocol establishes corresponding transfusion thresholds for critically ill and non-critically ill preterm infants based on their postnatal age in days. The details are as follows:

**Table:** 

Postnatal age (days)	Hb (g/L)	Hct (%)
≤7	120 (140)	35 (41)
8–21	105 (125)	31 (37)
>21	95 (115)	28 (34)

The values in parentheses represent a modest relaxation of the lower thresholds applicable to a minority of critically ill patients. Specific conditions include:

1. Invasive mechanical ventilation.
2. CPAP with FiO₂ > 25%, required for > 12 h/day.
3. Hemodynamically significant patent ductus arteriosus requiring treatment.
4. More than 6 episodes of apnea per day requiring stimulation, or more than 4 episodes of hypoxemia per day (SpO₂ < 60%), despite appropriate management.
5. Acute sepsis or necrotizing enterocolitis with circulatory failure requiring inotropic and/or vasopressor support.
6. Major surgery or significant bleeding (estimated blood loss > 10% of total blood volume).
7. Unexplained lactic acidosis (arterial lactate ≥ 4 mmol/L).

### 2.5 Administration

According to the Recommendations for prevention and treatment of vitamin D deficiency and vitamin D deficiency rickets issued by the National Scientific Research Collaborative Group for the Prevention and Treatment of Rickets (14), it is advised that preterm infants, low birth weight infants, and multiples receive vitamin D supplementation at 800–1000 IU/day soon after birth. In addition, the Expert consensus on clinical application of vitamin A and vitamin D in Chinese children (15) recommends oral vitamin A supplementation at 1500–2000 IU/day for the same population. Accordingly, all preterm infants included in this study were administered one capsule of vitamin AD drops daily (each capsule containing 1,500 IU vitamin A and 500 IU vitamin D; manufacturer: Qingdao Shuangjing Pharmaceutical Co., Ltd.) along with one capsule of vitamin D drops (400 IU vitamin D per capsule) until discharge. The specific postnatal day for initiating supplementation was determined by clinicians based on comprehensive assessment of individual risk factors. Serum 25-hydroxyvitamin D levels were monitored during hospitalization.

### 2.6 Statistical analysis

Statistical analyses were performed using SPSS 26.0 and R 4.5.0. Categorical variables were expressed as frequency and percentage [n (%)]. Continuous variables following a normal distribution were presented as mean ± standard deviation (Mean ± SD), while those with non-normal distribution were summarized as median and interquartile range [M (IQR)]. The correlation between the timing of vitamin AD initiation and the number of blood transfusions was assessed using Spearman’s rank correlation analysis. To account for the influence of the hospitalization timeline, segmented Poisson regression was employed to compare the incidence rate ratio (IRR) of blood transfusions before and after vitamin AD supplementation. Additionally, a conditional Cox proportional hazards model (Anderson-Gill model) was applied to evaluate the impact of vitamin AD supplementation on the risk of subsequent transfusions in preterm infants, with adjustments for vitamin D supplementation, oral iron therapy, hospitalization duration, and other covariates.

Based on the timing of vitamin AD initiation, infants were categorized into two groups: ≤14 days group and >14 days group. Propensity score matching was utilized to balance potential confounders between groups. Normally distributed continuous variables were compared using independent samples t-test; non-normally distributed continuous variables were analyzed with the Mann–Whitney U test; and categorical variables were compared using Pearson’s chi-square test. A generalized linear model (GLM) was applied to identify independent factors influencing the number of transfusions.

All generalized linear models were optimized via bidirectional variable selection. A two-sided *p*-value of less than 0.05 was considered statistically significant for all tests.

### 2.7 Sample size and statistical power

As a retrospective cohort study, all consecutive eligible infants (*N* = 226) were included. To assess the adequacy of this sample size, a post-hoc power analysis was performed using R 4.5.0.

For the primary analysis (multivariable negative binomial generalized linear model), a simulation-based power calculation was conducted using the observed effect size (*β* = 0.016 for the timing of vitamin AD initiation) and the final model parameters. This analysis indicated a statistical power >85% (*α* = 0.05). For the longitudinal analysis (conditional Cox model, Anderson-Gill model), Monte Carlo simulation based on the observed time-dependent hazard ratio (HR per log(day+1) = 0.381) yielded a statistical power >90%. Both post-hoc power estimates exceed the conventional 80% threshold, confirming that the sample size was sufficient for the study objectives.

## 3 Results

### 3.1 Clinical characteristics of included patients

Among the 226 preterm infants included, 133 (58.8%) were male. The median gestational age was 28.9 weeks (IQR: 27.4–30.3), and the median birth weight was 1,140 g (IQR: 910.0–1312.5). The median hemoglobin concentration at birth was 158 g/L (IQR: 140–170), with a median hematocrit of 47% (IQR: 43–51%). Regarding complications, patent ductus arteriosus was the most common (74.3%), while necrotizing enterocolitis and major gastrointestinal surgery were rare (both <3%). The median time to initiate enteral feeding was 1.9 days (IQR: 1.6–2.6), and the median time to achieve full enteral feeding was 21.4 days (IQR: 15.4–29.4). Oral iron supplementation was started at a median of 22 days (IQR: 17–33), with a median dose of 3.2 mg/kg (IQR: 2.9–3.6). During hospitalization, the median number of erythropoietin (EPO) administrations was 21 (IQR: 16–30), and the median dose per administration was 285.7 IU/kg (IQR: 262.1–315.6).

Vitamin AD supplementation was initiated at a median of 12 days after birth (IQR: 9–17), while vitamin D supplementation began at a median of 11 days (IQR: 9–14). In 181 infants, both supplements were started on the same day; the remaining 45 infants received vitamin D supplementation prior to vitamin AD. The median serum 25-hydroxyvitamin D concentration during hospitalization was 19 ng/mL (IQR: 16–21.25), with a peak concentration of 22 ng/mL (IQR: 19–26). The median total number of blood transfusions during hospitalization was 2 (IQR: 1–4; range: 1–14). Prior to vitamin AD supplementation, the median transfusion count was 1 (IQR: 1–3; range: 1–13), while after supplementation it was 1 (IQR: 0–1; range: 0–6). Other clinical characteristics are detailed in Table 1.

**Table 1:** Baseline characteristics of the study preterm infants.

Characteristic	*n* (%) or Median (IQR)
Male sex[n (%)]	133(58.8%)
Asphyxia[n (%)]	38(16.8%)
SGA[n (%)]	25(11.1%)
PDA[n (%)]	168(74.3%)
ICH[n (%)]	35(15.5%)
Grade I	21(9.3%)
Grade II	10(4.4%)
Grade III	3(1.3%)
Grade IV	1(0.4%)
FI[n (%)]	31(13.7%)
GI intolerance[n (%)]	27(11.9%)
NEC[n (%)]	5(2.2%)
Major GI surgery[n (%)]	4(1.8%)
Cholestasis[n (%)]	21(9.3%)
Sepsis[n (%)]	97(42.9)
BPD[n (%)]	122(54.0%)
ROP[n (%)]	58(25.7%)
MV[n (%)]	144(63.7%)
LOS[Median (IQR), days]	63.4(51.7–77.4)
GA[Median (IQR), weeks]	28.9(27.4–30.3)
BW[Median (IQR), g]	1140.0(910.0–1312.5)
APGAR-1[Median (IQR)]	8(8–10)
APGAR-5[Median (IQR)]	10(9–10)
HCT at birth[Median (IQR), %]	47(43–51)
Hb at birth[Median (IQR), g/L]	158(140–170)
Breastfeeding initiation time[Median (IQR), d]	1.9(1.6–2.6)
Time to full enteral feeding[Median (IQR), d]	21.4(15.4–29.4)
Oral iron initiation time[Median (IQR), d]	22(17–33)
Iron supplementation dose[Median (IQR), mg/kg]	3.2(2.9–3.6)
EPO administrations	21(16–30)
EPO dose[Median (IQR), IU/kg]	285.7(262.1–315.6)
Time to VitAD initiation[Median (IQR), days]	12(9–17)
Time to VitD initiation[Median (IQR), days]	11(9–14)
Mean 25(OH)D (hospitalization) [Median (IQR), ng/ml]	19(16—21.25)
Peak 25(OH)D (hospitalization)[Median (IQR), ng/ml]	22(19–26)
Total transfusions[Median (IQR)]	2(1–4) Min = 1 Max = 14
Transfusions pre-VitAD[Median (IQR)]	1(1–3) Min = 0 Max = 13
Transfusions post-VitAD[Median (IQR)]	1(0–1) Min = 0 Max = 6

Data are presented as n (%), Mean ± SD, or Median (IQR) as appropriate. SGA, small for gestational age; PDA, patent ductus arteriosus; ICH, intracranial hemorrhage; FI, feeding intolerance; GI, gastrointestinal; NEC, necrotizing enterocolitis; BPD, bronchopulmonary dysplasia; ROP, retinopathy of prematurity; MV, mechanical ventilation; LOS, length of hospital stay; GA, gestational age; BW, birth weight; VitAD, vitamin AD; VitD, vitamin D; IQR, interquartile range; APGAR-1, APGAR score at 1 min; APGAR-5, APGAR score at 5 min; Hb, Hemoglobin; HCT, hematocrit; EPO, Erythropoietin; 25(OH)D, 25-Hydroxyvitamin D.

### 3.2 Outcome measures

#### 3.2.1 Association between timing of vitamin AD and vitamin D supplementation and number of blood transfusions

**Figure 1:**
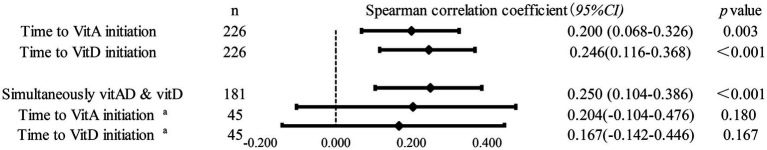
Correlation between the timing of vitamin AD / vitamin D supplementation and the number of blood transfusions. Among the 45 children who received vitamin D supplementation followed by vitamin AD.

In the overall cohort, the timing of vitamin AD initiation was significantly positively correlated with the number of blood transfusions [Spearman’s correlation coefficient = 0.200(0.068–0.326), *p* = 0.003]. Similarly, a significant positive correlation was observed between the initiation of vitamin D supplementation and transfusion frequency [Spearman’s correlation coefficient = 0.246(0.116–0.368), *p* < 0.001]. Among the 181 infants who started vitamin AD and vitamin D supplementation simultaneously, the timing of supplementation remained positively correlated with transfusion count [Spearman’s correlation coefficient = 0.246(0.116–0.368), *p* < 0.001]. In contrast, no significant correlation was found between the timing of either vitamin A or vitamin D supplementation and the number of transfusions in the subgroup of 45 infants who received vitamin D prior to vitamin AD. Given that most infants received both supplements concurrently and both showed significant effects, the timing of vitamin AD supplementation was used as a unified exposure variable in subsequent analyses (Figure 1).

**Figure 2:**
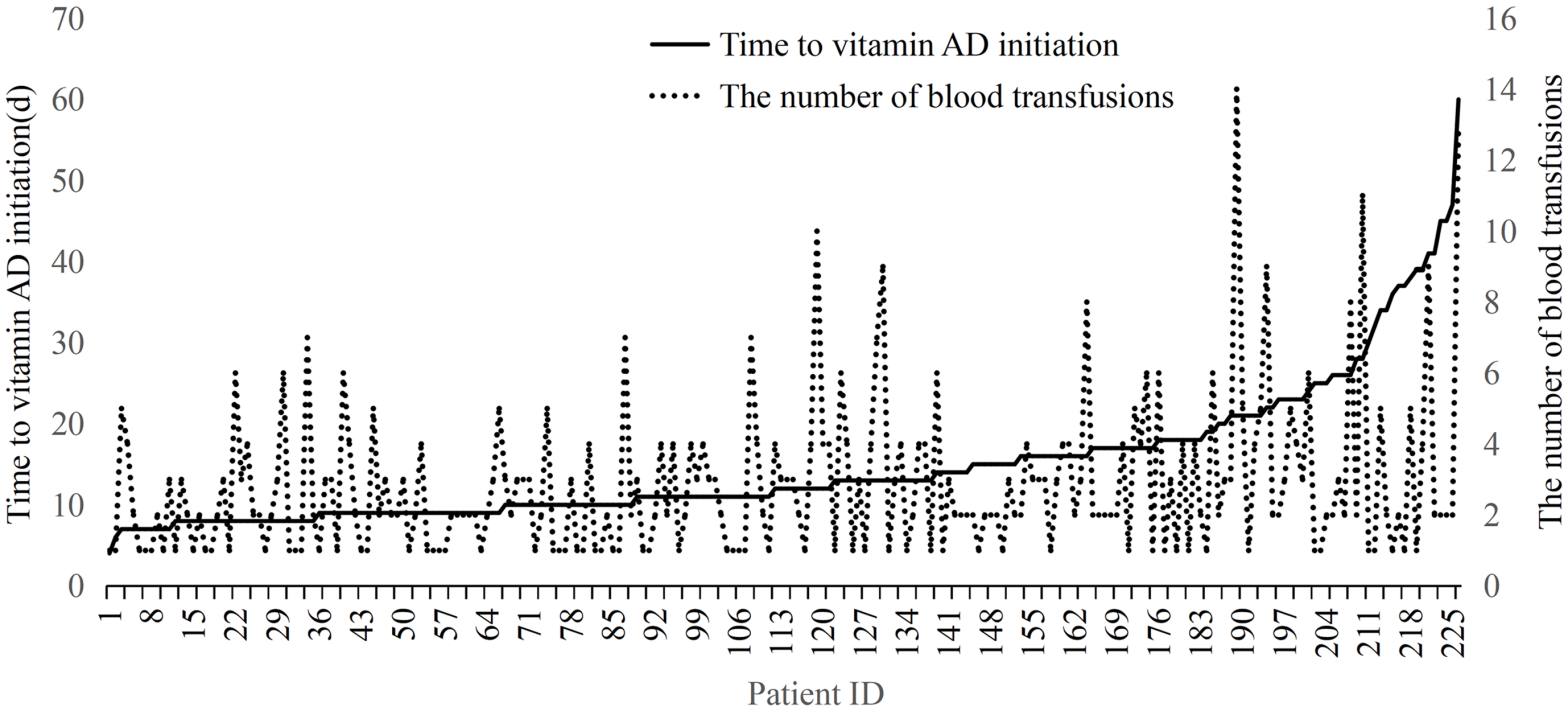
Relationship between the timing of vitamin AD initiation and the number of blood transfusions.

**Figure 3:**
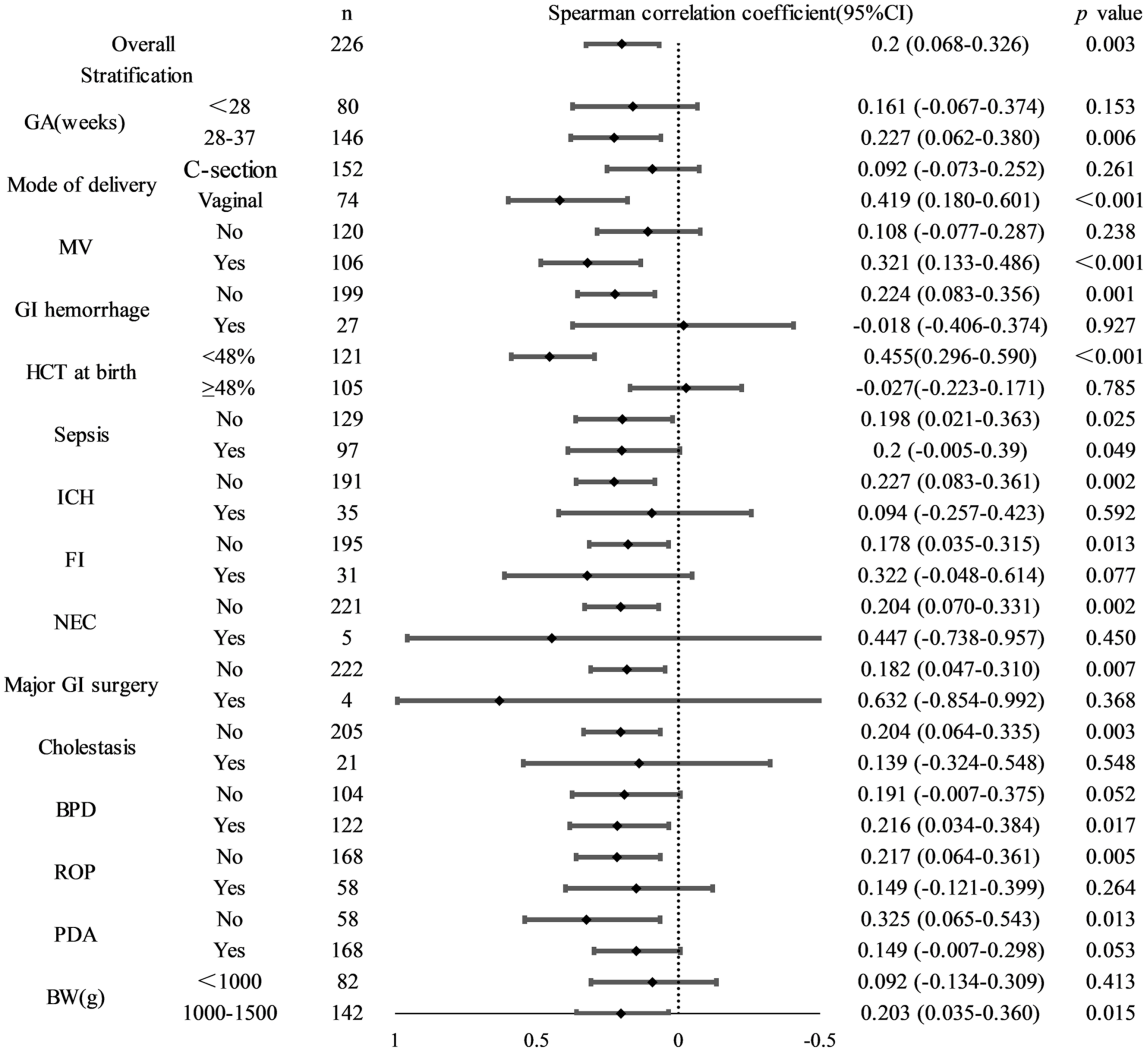
Stratified correlation analysis between timing of vitamin AD supplementation and number of blood transfusions.

Furthermore, an increased delay in the initiation of vitamin AD supplementation was associated with a higher number of blood transfusions (Figure 2). Spearman’s rank correlation analysis confirmed a significant positive correlation between the timing of vitamin A initiation and transfusion frequency in the overall cohort [*ρ* = 0.200(0.068–0.326), *p* = 0.003]. This association remained statistically significant (*p* < 0.05) in several predefined subgroups, including infants with gestational ages between 28 and 36^+6^ weeks, those delivered vaginally, those receiving mechanical ventilation, those without complications such as gastrointestinal hemorrhage or sepsis, those with birth weights between 1,000 and 1,500 g, and those with HCT at birth of <48%(Figure 3).

The multivariable negative binomial generalized linear model including all potential confounders initially showed that the timing of vitamin AD initiation was not an independent factor influencing transfusion frequency [*β* = 0.010(−0.005 to 0.024), *p* = 0.180]. However, following bidirectional variable selection for model optimization, the overall model fit improved substantially: the Omnibus test *p*-value increased from 0.136 to < 0.001, and the Akaike Information Criterion (AIC) decreased from 855.16 to 641.77. The final optimized model demonstrated that the timing of vitamin AD initiation was an independent risk factor for transfusion frequency [*β* = 0.016(0.007 to 0.025), *p* < 0.001; Table 2].

**Table 2:** Independent risk factors for transfusion frequency.

Model parameters	Full model			Step model (Direction = Both)		
	Regression coefficient *β* (95%*CI*)	Wald χ^2^	*p* value	Regression coefficient β (95%*CI*)	Wald χ^2^	*p* value
Intercept	1.046(−1.159 to 3.250)	0.86	0.35	2.306(1.449 to 3.154)	28.08	0.00
Time to vitamin A initiation(d)	0.010(−0.005 to 0.024)	1.72	0.19	0.016(0.007 to 0.025)	12.38	0.00
Male	0.066(−0.071 to 0.203)	0.90	0.34			
Multiple birth	0.149(0.000 to 0.298)	3.83	0.05			
Cesarean delivery	−0.218(−0.387 to −0.049)	6.41	0.01	−0.278(−0.472 to −0.082)	7.86	0.01
Asphyxia	−0.016(−0.233 to 0.202)	0.02	0.89			
SGA	−0.199(−0.464 to 0.065)	2.19	0.14			
PDA	−0.080(−0.236 to 0.075)	1.03	0.31			
ICH	0.113(−0.100 to 0.327)	1.08	0.30			
FI	−0.209(−0.400 to −0.017)	4.58	0.03			
GI hemorrhage	0.219(0.024 to 0.413)	4.87	0.03			
NEC	−0.324(−0.741 to 0.093)	2.31	0.13			
Major GI surgery	0.081(−0.478 to 0.640)	0.08	0.78			
Cholestasis	−0.010(−0.242 to 0.222)	0.01	0.93			
Sepsis	0.080(−0.067 to 0.228)	1.13	0.29			
BPD	0.104(−0.058 to 0.265)	1.59	0.21			
ROP	0.100(−0.068 to 0.267)	1.36	0.24			
MV	0.202(0.052 to 0.352)	7.01	0.01	0.250(0.047 to 0.459)	5.69	0.02
GA(weeks)	0.040(−0.043 to 0.122)	0.90	0.34			
BW(g)	−0.001(−0.002 to −0.001)	14.96	0.00	−0.001(−0.001 to 0.000)	16.09	0.00
APGAR-1	−0.035(−0.089 to 0.019)	1.61	0.20			
APGAR- 5	0.048(−0.018 to 0.113)	2.04	0.15			
Breastfeeding initiation time(d)	0.093(0.039 to 0.148)	11.11	0.00	0.091(0.028 to 0.151)	8.50	0.00
Time to full enteral feeding(d)	0.010(0.003 to 0.018)	7.42	0.01	0.008(0.000 to 0.017)	3.80	0.05
Vitamin D lead time(d)	0.189(−0.016 to 0.021)	0.08	0.77			
Mean 25(OH)D(ng/ml)	−0.008(−0.024 to 0.009)	0.78	0.38			
Hb at birth(g/L)	−0.001(−0.008 to 0.006)	0.10	0.76	−0.007(−0.012 to −0.003)	11.72	0.00
HCT at birth(%)	−0.015(−0.039 to 0.010)	1.32	0.25			
EPO administrations	−0.004(−0.011 to 0.004)	0.87	0.35			
Oral iron initiation time(d)	0.005(0.001 to 0.008)	5.65	0.02	0.004(−0.001 to 0.008)	2.82	0.09
	Omnibus test: *p* = 0.136			Omnibus test: *p*<0.001		
	AIC: 855.16			AIC: 641.77		

SGA, small for gestational age; PDA, patent ductus arteriosus; ICH, intracranial hemorrhage; FI, feeding intolerance; GI, gastrointestinal; NEC, necrotizing enterocolitis; BPD, bronchopulmonary dysplasia; ROP, retinopathy of prematurity; MV, mechanical ventilation; LOS, length of hospital stay; GA, gestational age; BW, birth weight; VitAD, vitamin AD; VitD, vitamin D; IQR, interquartile range; APGAR-1, APGAR score at 1 min; APGAR-5, APGAR score at 5 min; Vitamin D lead time, Lead time of vitamin D supplementation (days before vitamin AD initiation); Hb, Hemoglobin; HCT, hematocrit; EPO, Erythropoietin; 25(OH)D, 25-Hydroxyvitamin D.

#### 3.2.2 Effect of vitamin AD supplementation on subsequent transfusions

**Figure 4:**
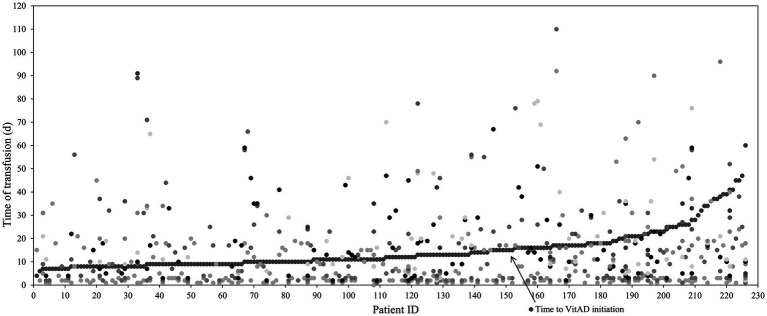
Temporal distribution of transfusions before and after vitamin AD supplementation.

The distribution of transfusion timing differed before and after vitamin AD supplementation (Figure 4). Segmented Poisson regression analysis, adjusted for the hospitalization timeline, showed that the incidence rate ratio (IRR) for transfusions during the post-supplementation period was 0.139 (0.098–0.199; Table 3).

**Table 3:** Incidence rate ratio (IRR) of transfusion before and after vitamin AD supplementation.

Model parameters	*IRR*(95%CI)	*p* value
Intercept	0.134(0.120–0.149)	<0.001
VitA status	0.139(0.098–0.199)	<0.001
Days since admission	0.995(0.972–1.017)	0.680

**Figure 5:**
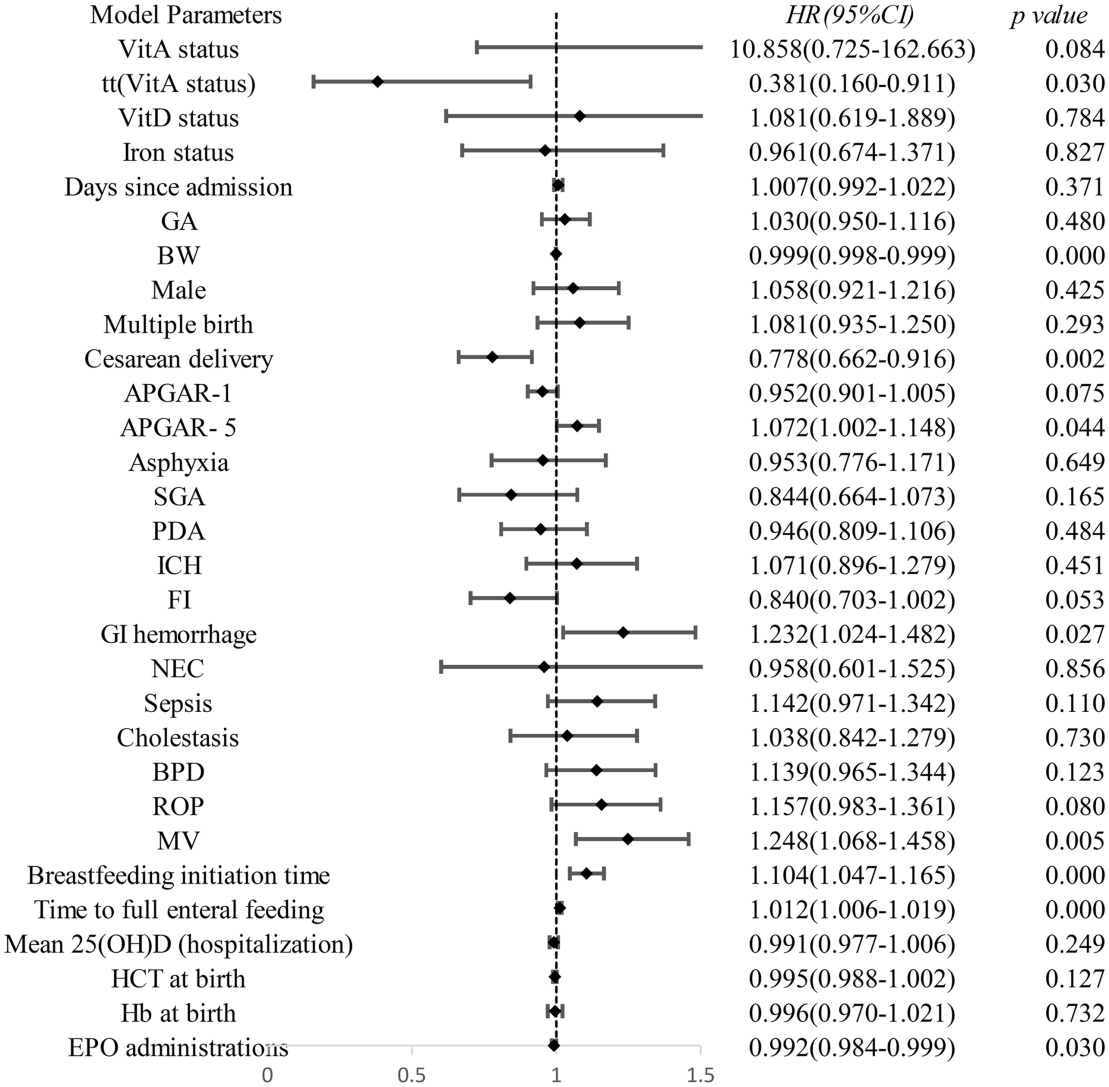
Association between vitamin AD supplementation and the risk of subsequent transfusions.

**Figure 6:**
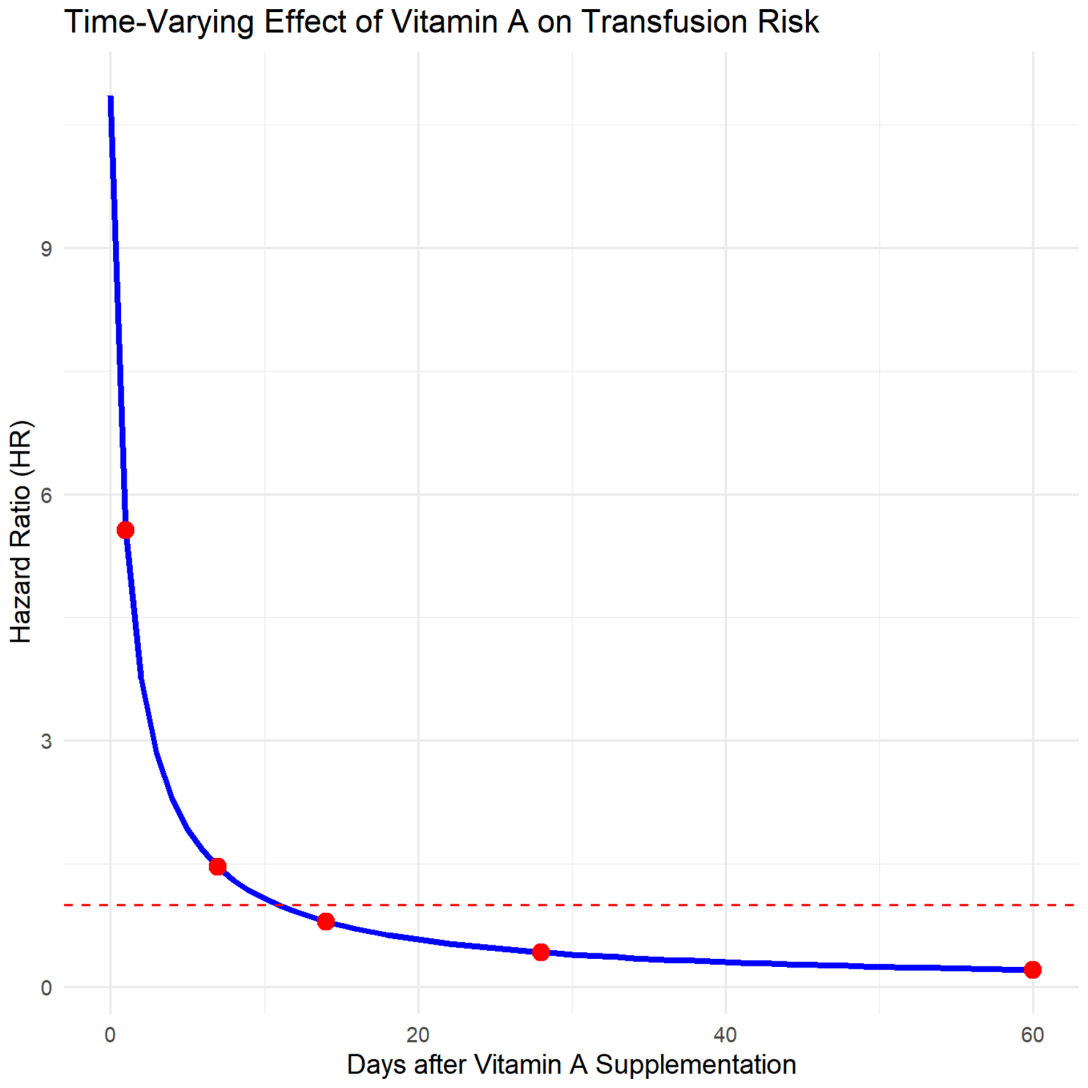
Time-varying effect of vitamin A on transfusion risk.

The conditional Cox proportional hazards model (Anderson-Gill model), adjusted for vitamin D supplementation, oral iron therapy, hospitalization timeline, and other covariates, showed no immediate significant change in the risk of subsequent transfusion events after vitamin AD supplementation in preterm infants [*HR* = 10.858(0.725–162.663), *p* = 0.084]. However, a time-dependent protective effect was observed [*HR* = 0.381(0.160–0.911), *p* = 0.03; Figure 5]. The time-varying effect of vitamin A supplementation on transfusion risk is presented in Figure 6. The hazard ratio transitioned from a non-significant initial value to a protective one, with the effect turning point estimated at approximately 5 days post-supplementation. By day 28, the modeled HR was 0.32, corresponding to an estimated 68% reduction in transfusion risk.

#### 3.2.3 Group comparison

Based on the initiation timing of vitamin AD supplementation, patients were categorized into two groups: ≤14 days (*n* = 144) and >14 days (*n* = 82). After propensity score matching was performed to balance covariates including time to full enteral feeding, hemoglobin concentration at birth, and time of oral iron initiation, 58 patients were successfully matched in a 1:1 ratio. Following matching, no statistically significant difference was observed in the number of blood transfusions between the two groups [median (IQR): 2 (1.5–3) vs. 2 (2–4), *p* = 0.544; Table 4].

**Table 4:** Transfusion frequency after propensity score matching in groups defined by vitamin AD initiation timing.

Clinical features	Pre-propensity score			Post-propensity score		
	Time to vitamin A initiation ≤14d (*n* = 144)	Time to vitamin A initiation >14d (*n* = 82)	*p* value	Time to vitamin A initiation ≤14d (*n* = 29)	Time to vitamin A initiation >14d (*n* = 29)	*p* value
Male	80(55.56%)	53(64.63%)	0.182	16(55.2%)	17(58.6%)	0.791
Multiple birth	55(38.19%)	28(34.15%)	0.544	9(31.0%)	14(48.3%)	0.180
Cesarean delivery	100(69.44%)	52(63.41%)	0.579	19(65.5%)	19(65.5%)	1.000
Asphyxia	24(16.67%)	14(17.07%)	0.937	6(20.7%)	6(20.7%)	1.000
SGA	15(10.42%)	10(12.20%)	0.682	5(17.2%)	1(3.4%)	0.196
PDA	102(70.83%)	66(80.49%)	0.110	21(72.4%)	22(75.9%)	0.764
ICH	22(15.3%)	13(15.9%)	0.502	4(13.8%)	3(10.3%)	0.687
FI	17(11.81%)	14(17.07%)	0.268	3(10.3%)	5(17.2%)	0.703
GI hemorrhage	15(10.42%)	12(14.63%)	0.347	5(17.2%)	5(17.2%)	1.000
NEC	2(1.39%)	3(3.66%)	0.356	1(3.4%)	1(3.4%)	1.000
Major GI surgery	1(0.69%)	3(3.66%)	0.137	0(0.0%)	0(0.0%)	1.000
Cholestasis	14(9.72%)	7(8.54%)	0.768	3(10.3%)	4(13.8%)	0.687
Sepsis	61(42.36%)	36(43.90%)	0.822	15(51.7%)	11(37.9%)	0.291
BPD	80(55.56%)	42(51.22%)	0.529	15(51.7%)	16(55.2%)	0.792
ROP	37(25.69%)	21(25.61%)	0.989	6(20.7%)	7(24.1%)	0.753
MV	68(47.22%)	38(46.34%)	0.898	17(58.6%)	18(62.1%)	0.788
GA(weeks)	28.9 ± 1.9	28.6 ± 2.0	0.425	28.28 ± 1.40	28.39 ± 1.84	0.784
BW(g)	1150.0(930.0–1327.5)	1090.0(895.0–1292.5)	0.368	1,120(910–1,350)	1,050(905–1,230)	0.460
APGAR-1	8.0(7.3–10.0)	8(8–10)	0.842	8(7–10)	8(7–9)	0.766
APGAR- 5	9.5(9.0–10.0)	10(9–10)	0.427	10(9–10)	10(9–10)	0.782
Breastfeeding initiation time(d)	3(2–3)	2(2–4)	0.589	3.0(2–3.5)	2(2–4)	0.915
Time to full enteral feeding(d)	20.5(15–29)	26(17–34)	0.027	21(15.5–36.5)	28(17–33)	0.827
Vitamin D lead time(d)	0(0–0)	0(0–11.75)	<0.001	0(0–0)	0(0–0)	0.643
Mean 25(OH)D (ng/ml)	19(17–22)	18(16–21)	0.184	18.17 ± 3.07	18.17 ± 3.69	0.837
Hb at birth(g/L)	152.74 ± 20.02	159.79 ± 22.58	0.016	159(141.5–167)	159(148–171.5)	0.549
HCT at birth(%)	47(42–50)	49(44.75–52.53)	0.215	47.1(42–51.95)	48.5(45–52.8)	0.446
EPO administrations	22.47 ± 10.86	20.72 ± 10.48	0.241	20.76 ± 9.66	21.28 ± 9.40	0.719
Oral iron initiation time(d)	20.5(16–31)	25(18–34)	0.036	19(16–28)	25(18.5–29.5)	0.170
The number of blood transfusions	2(1–3)	2(2–4)	0.031	2(1.5–3)	2(2–4)	0.544

Data are presented as n (%), Mean ± SD, or Median (IQR) as appropriate. Abbreviations: SGA, small for gestational age; PDA, patent ductus arteriosus; ICH, intracranial hemorrhage; FI, feeding intolerance; GI, gastrointestinal; NEC, necrotizing enterocolitis; BPD, bronchopulmonary dysplasia; ROP, retinopathy of prematurity; MV, mechanical ventilation; LOS, length of hospital stay; GA, gestational age; BW, birth weight; VitAD, vitamin AD; VitD, vitamin D; IQR, interquartile range; APGAR-1, APGAR score at 1 min; APGAR-5, APGAR score at 5 min; Hb, Hemoglobin; HCT, hematocrit; EPO, Erythropoietin; Vitamin D lead time, Lead time of vitamin D supplementation (days before vitamin AD initiation); 25(OH)D, 25-Hydroxyvitamin D.

In the multivariable negative binomial generalized linear model, the timing of vitamin AD initiation within 14 days after birth showed no independent association with transfusion frequency [*β* = −0.042(−0.216 to 0.133), *p* = 0.640]. Following bidirectional variable selection for model optimization, this variable remained a non-significant independent factor [*β* = −0.010(−0.218 to 0.202), *p* = 0.930; Table 5].

**Table 5:** Independent risk factors for transfusion frequency by two groups.

Model parameters	Full model			Step model (direction = both)		
	Regression coefficient β (95%CI)	Wald χ^2^	*p* value	Regression coefficient β (95%CI)	Wald χ^2^	*p* value
Intercept	1.402(−0.712 to 3.517)	1.69	0.19	2.486(1.592 to 3.373)	29.95	0.00
Time to vitamin A initiation ≤14d	−0.042(−0.216 to 0.133)	0.22	0.64	−0.010(−0.218 to 0.202)	0.01	0.93
Male	0.064(−0.074 to 0.203)	0.83	0.36			
Multiple birth	0.153(0.002 to 0.304)	3.97	0.05			
Cesarean delivery	−0.214(−0.383 to −0.044)	6.09	0.01	−0.289(−0.483 to −0.093)	8.48	0.00
Asphyxia	0.001(−0.216 to 0.218)	0.00	0.99			
SGA	−0.174(−0.429 to 0.080)	1.80	0.18			
PDA	−0.081(−0.239 to 0.076)	1.02	0.31			
ICH	0.112(−0.103 to 0.327)	1.05	0.31			
FI	−0.219(−0.412 to −0.027)	5.00	0.03			
GI hemorrhage	0.238(0.043 to 0.434)	5.74	0.02	0.205(−0.060 to 0.456)	2.43	0.12
NEC	−0.346(−0.758 to 0.066)	2.71	0.10			
Major GI surgery	0.117(−0.444 to 0.679)	0.17	0.68			
Cholestasis	−0.024(−0.253 to 0.205)	0.04	0.84			
Sepsis	0.097(−0.050 to 0.244)	1.69	0.19			
BPD	0.100(−0.064 to 0.264)	1.44	0.23			
ROP	0.230(−0.085 to 0.253)	0.96	0.33			
MV	0.197(0.048 to 0.345)	6.75	0.01	0.225(0.018 to 0.438)	4.43	0.04
GA(weeks)	0.032(−0.050 to 0.113)	0.58	0.45			
BW(g)	−0.001(−0.002 to −0.001)	14.65	0.00	−0.001(−0.001 to −0.001)	17.18	0.00
APGAR-1	−0.031(−0.085 to 0.024)	1.22	0.27			
APGAR- 5	0.048(−0.018 to 0.113)	2.04	0.15			
Breastfeeding initiation time(d)	0.095(0.039 to 0.151)	10.99	0.00	0.105(0.041 to 0.167)	10.80	0.00
Time to full enteral feeding(d)	0.011(0.003 to 0.018)	7.85	0.01	0.009(0.000 to 0.017)	4.35	0.04
Time to vitamin D initiation(d)	0.012(−0.001 to 0.024)	3.22	0.07	0.016(0.003 to 0.028)	6.11	0.01
Mean 25(OH)D(ng/ml)	−0.009(−0.025 to 0.007)	1.15	0.28			
Hb at birth(g/L)	−0.001(−0.008 to 0.006)	0.07	0.79	−0.007(−0.011 to −0.003)	10.63	0.00
HCT at birth(%)	−0.015(−0.041 to 0.012)	1.22	0.27			
EPO administrations	−0.004(−0.012 to 0.004)	0.96	0.33			
Oral iron initiation time(d)	0.005(0.001 to 0.009)	5.74	0.02	0.004(−0.001 to 0.008)	3.17	0.07
	Omnibus test: *p* = 0.141			Omnibus test: *p*<0.001		
	AIC: 855.36			AIC: 646.56		

Data are presented as n (%), Mean ± SD, or Median (IQR) as appropriate. LOS, length of hospital stay; NEC, necrotizing enterocolitis; FI, feeding intolerance.

## 4 Discussion

This retrospective study identified a statistical association between the timing of vitamin AD supplementation initiation and the number of transfusions received by preterm infants during hospitalization. In multivariable modeling, the timing of supplementation emerged as an independent factor influencing transfusion frequency, and longitudinal analyses suggested a potential time-dependent protective effect following administration. However, when early supplementation was analyzed as a dichotomous variable using a 14-day cutoff, the difference in transfusion burden between groups did not reach statistical significance after propensity score matching. Furthermore, in subsequent multivariable models, this grouping factor did not demonstrate an independent association with transfusion frequency. Collectively, these findings point to a complex relationship between the timing of vitamin AD supplementation and transfusion requirements in preterm infants, warranting cautious interpretation of its clinical significance.

Our findings align with previous studies demonstrating the beneficial role of micronutrient supplementation in improving anemia, while extending the evidence through its focus on timing, combination therapy, and a clinically relevant outcome. Several randomized controlled trials in infants have indicated that multiple micronutrient supplementation—including vitamins A and D—can effectively enhance hematological and micronutrient status (16–18). For example, Cardoso et al. (17) observed significantly lower rates of anemia and vitamin A deficiency among infants who received micronutrient-fortified complementary foods. Similarly, Hop and Berger (18) reported that daily micronutrient supplementation was more effective than placebo or weekly supplementation in improving hemoglobin and iron status. Although these studies involved older infants and used multi-micronutrient interventions, they consistently support the value of micronutrients—including vitamins A and D—in anemia prevention and treatment.

The present study extends this evidence base by focusing specifically on preterm infants—a population uniquely vulnerable to micronutrient deficiency due to limited intrauterine stores and increased postnatal demands—and by examining a clinically meaningful hard endpoint: transfusion requirements. In this context, our results are consistent with those reported by Chai et al. (19) in preterm infants. That study demonstrated that combining vitamin A and vitamin B with recombinant human erythropoietin significantly reduced transfusion rates compared to single-vitamin regimens, suggesting a potential synergistic or additive effect of vitamin A. Our study strengthens and refines this observation by indicating that early prophylactic vitamin AD supplementation may confer transfusion-related benefits, and by providing an initial exploration of the impact of supplementation timing on this effect.

The mechanisms underlying these effects are likely multifactorial, involving synergistic roles of vitamins A and D in the regulation of erythropoiesis and iron metabolism. It must be emphasized, however, that the present study did not measure key biomarkers such as ferritin, serum iron, total iron-binding capacity, or erythropoietin levels. Consequently, while the proposed mechanisms—including improved iron utilization, enhanced endogenous erythropoietin responsiveness, and anti-inflammatory effects—provide a compelling biological framework based on the existing literature, they remain speculative in the context of our findings and cannot be empirically validated by the current data.

From a biological perspective, vitamin A, via its active metabolite retinoic acid, binds to nuclear retinoic acid receptors (RAR/RXR), forming heterodimers that interact with retinoic acid response elements on target genes to finely regulate the expression of a cascade of genes involved in hematopoiesis. During embryonic development, it helps regulate the endothelial-hematopoietic balance in the yolk sac and activates definitive hematopoietic programs in the aorta-gonad-mesonephros region. Postnatally, vitamin A not only stimulates renal erythropoietin production but also maintains the homeostasis and self-renewal capacity of the hematopoietic stem cell pool (20). More importantly, vitamin A deficiency has been shown to impair duodenal iron absorption, interfere with iron mobilization from the reticuloendothelial system, and affect iron transport and utilization by erythroid precursors, leading to functional iron deficiency and anemia despite adequate iron stores—a phenomenon bearing resemblance to the aberrant iron metabolism observed in the “anemia of inflammation” commonly seen in preterm infants (21). The reduction in transfusion frequency following vitamin AD supplementation observed in our study may partially reflect the corrective effect of vitamin A on dysregulated iron metabolism. Additionally, the immunomodulatory properties of vitamin A, particularly its influence on T-cell differentiation and cytokine profiles, may help mitigate the suppression of erythropoiesis induced by chronic inflammatory states arising from infection, necrotizing enterocolitis, or surgical stress (22).

The role of vitamin D is equally critical and may interact with that of vitamin A. Its main active form, 1,25-dihydroxyvitamin D₃ [1,25-(OH)₂D₃], not only directly regulates gene expression in the hematopoietic microenvironment by binding to the vitamin D receptor, promoting the proliferation and differentiation of erythroid burst-forming units (BFU-E) and erythroid colony-forming units (CFU-E), but has also been shown to act synergistically with erythropoietin both *in vitro* and *in vivo* (23). Epidemiological studies suggest that maintaining serum 25(OH)D levels between 59.7 and 70.3 nmol/L is independently associated with a lower risk of anemia (24). Vitamin D may also promote erythropoiesis through several indirect pathways: it suppresses the secretion of parathyroid hormone (a negative regulator of erythropoiesis) and downregulates fibroblast growth factor-23 levels, thereby improving bone marrow microenvironment responsiveness to erythropoietin (25). Previous research has indicated that 25(OH)D deficiency is significantly associated with an increased risk of anemia in children (26), and vitamin D nutritional status is positively correlated with erythrocyte parameters and iron metabolism markers such as mean corpuscular volume, serum ferritin, and transferrin saturation (27).

Although the pathophysiological mechanisms linking vitamin D deficiency to anemia—such as direct effects on iron metabolism, inhibition of erythroid precursor proliferation, and modulation of inflammatory pathways—have been relatively well described, high-quality evidence regarding the impact of the timing of vitamin D (particularly in combination with vitamin A) supplementation on transfusion outcomes in preterm infants remains limited. Our observation that later supplementation is associated with a higher number of transfusions aligns with the concept of a “critical window” in preterm infant developmental biology: the notion that providing essential nutrients during specific periods of rapid development and maturation of certain organ systems (including the hematopoietic system) may yield maximal long-term benefits. This hypothesis is further supported by our segmented Poisson regression analysis, which demonstrated a significant and sustained reduction in the incidence rate of transfusions following the initiation of vitamin AD supplementation; moreover, the conditional Cox model revealed a time-dependent protective effect. Notably, the model indicated that the protective effect became apparent and persisted approximately 5 days after supplementation initiation. This time lag aligns reasonably with the physiological processes required for the absorption of these fat-soluble vitamins in the small intestine, their storage and metabolic hydroxylation in the liver, and their eventual distribution to target cells in the bone marrow to initiate a cascade of gene expression and protein synthesis.

However, it must be acknowledged that not all studies in the literature have reported positive outcomes, and these inconsistencies provide important context for interpreting our data. For instance, a large randomized trial by Edmond et al. (28) found that a single high dose of vitamin A supplementation administered during the neonatal period (400,000 IU to mothers or 50,000 IU to neonates weighing >1,500 g) failed to significantly improve hemoglobin levels or reduce anemia prevalence at 6 months of age. These seemingly contradictory results may be attributable to key differences in intervention design, dosage regimen, and study population characteristics. First, preterm infants are born with extremely low hepatic vitamin A stores and, due to rapid postnatal growth and high demands, are at substantial risk for “preterm infant vitamin A deficiency.” For a population with such a persistent risk of deficiency, a single high-dose bolus may only transiently elevate serum levels and fail to provide sustained substrate support. In contrast, the daily low-dose supplementation regimen used in our study may more closely mimic the physiological pattern of continuous intrauterine supply, potentially maintaining more stable effective concentrations at target organs. Second, the choice of outcome measure differs: changes in short-term transfusion requirements may be more sensitive to nutritional interventions than distal hemoglobin levels, as they integrate dynamic changes in multiple factors during the acute phase of illness (e.g., iatrogenic blood loss, infection, inflammation).

Despite adherence to a restrictive blood-drawing policy and the use of microsampling techniques in clinical practice, a key methodological limitation of this study is the inability to precisely quantify the cumulative iatrogenic blood loss from frequent laboratory testing during hospitalization. Such blood loss is a well-established contributor to anemia in preterm infants. This unmeasured confounding variable may link greater illness severity—and the consequent delay in supplementation—with a higher transfusion requirement, potentially introducing bias into the observed association.

In addition to the specific issue of iatrogenic blood loss, the findings of this study should be interpreted within the context of several broader methodological limitations. First, the observational retrospective design precludes causal inference; we can only reveal associations. Although multivariable regression and propensity score matching were employed to control for known confounders, the possibility of residual confounding—particularly from unmeasured or difficult-to-quantify factors such as feeding tolerance, occult blood loss, or subtle variations in transfusion practices—remains. Second, the dichotomous grouping using a 14-day cutoff did not demonstrate an independent association with transfusion frequency after matching or in multivariable models. This may reflect that the relationship between supplementation timing and transfusion risk is not a simple linear threshold effect, but could also be related to loss of information from dichotomization and reduced statistical power post-matching. Third, the decision to initiate supplementation is itself influenced by clinical stability, introducing potential “confounding by indication.” Finally, as a single-center study with a relatively modest sample size and lacking biomarker data on iron metabolism and hematopoietic regulation, our ability to explore mediating mechanisms and generalize findings is limited.

In conclusion, this study identified a statistical association between the timing of vitamin AD supplementation and the number of transfusions in preterm infants, with earlier supplementation potentially linked to a lower transfusion burden and a time-dependent protective trend observed post-supplementation. This finding provides valuable clues for clinical practice and points the way for future research. Whether vitamin AD supplementation can serve as a safe and effective strategy to reduce anemia-related transfusions in preterm infants—and crucially, the optimal timing, most appropriate dosage, and target population most likely to benefit—must be further elucidated and validated through well-designed, adequately powered, large-scale prospective randomized controlled trials.

## 5 Conclusion

This study suggests that earlier initiation of vitamin AD supplementation is associated with a reduced frequency of blood transfusions in preterm infants, showing a time-dependent protective effect. However, no significant difference was found when comparing groups based on a 14-day cutoff. The findings highlight the potential benefit of timely supplementation, though the optimal timing requires further prospective research.

## Data Availability

The raw data supporting the conclusions of this article will be made available by the authors, without undue reservation.
